# Failure of Artificial Dentures under Modern Methods, and a Simple Remedy

**Published:** 1922-03

**Authors:** Stewart J. Spence

**Affiliations:** Chattanooga, Tennessee


### "FAILURE OF ARTIFICIAL DENTURES UNDER MODERN METHODS, AND A SIMPLE REMEDY"

BY STEWART J. SPENCE, CHATTANOOGA, TENNESSEE

In Dr. McLaren's article, which appeared in the *Dental Digest* of December, 1921, he suggests a method for preventing compression of cast in flask-closing, by using rubber so softened by benzine as not to require heavy pressure. He says that any good plaster of Paris may be used instead of the "artificial stones on the market." Permit me to make a few comments.

As all plaster of Paris expands in setting, Dr. McLaren's method does not provide against misfit from this source.

As all plaster of Paris (and some of the "artificial stones") become so softened during vulcanization as not to have sufficient strength to resist the effort of vulcanite to contract in cooling, therefore Dr. McLaren's investment of mere plaster of Paris is apt to result in shrunken and (probably) warped plates.

Several of the "artificial stones" set about fourfold harder than does plaster of Paris, and my experiments prove that this is sufficient hardness to prevent any appreciable compression of the cast in flask-closing. Were it not so, I should make my plaster to set harder; which can be done by the dentist by simply adding a retarder and working it longer. About ten grains of alum to a mix and ten minutes more of working it, fully doubles the hardness. But this is unnecessary, unless possibly for swaging metal plates by the hammer.

Dr. McLaren speaks of *often rebasing.* This is contrary to my experience. He also speaks of *regrinding,* rightly supposing that this may be needed by the teeth in flask-closing being forced into the investment. This is very apt to occur if mere plaster of Paris is used for the investment; but when any of the good "artificial stones" are used such compression is inappreciable. Perhaps, also, it occurs to some extent when, as advised by some high authorities, fifty per cent. only of Spence's Plaster is used in investing, the balance being plaster of Paris. I prefer to use it "straight." However, some regrinding is usually required, this probably being due to our not getting perfect occlusion while setting up the teeth; the shrinkage of the melted wax in cooling, and other things, making this well-nigh impossible.

Dr. McLaren mentions a cone-shaped space in the plaster investment being filled by swollen rubber. Of course we know the rubber will swell from heat, but this can occur only when it is not firmly held in on all sides. After vulcanization, when the investing plaster has become softened, the vulcanite will contract in cooling, unless firmly held by its investment; and mere plaster of Paris is rarely firm enough. I can not see how the rubber can expand in a tightly closed flask and an unsoftened investment, but it is easy to see how it can contract in a softened investment.

Like soft rubber, plaster of Paris expands when heated to about 300 degrees F., but like it, this can not occur in a firm investment; and pressure-hardened rubber is very firm. The cone-shaped space could have been filled by either or both of these swellings.—*Dental Digest.*

